# AVSS: Airborne Video Surveillance System

**DOI:** 10.3390/s18061939

**Published:** 2018-06-14

**Authors:** Jongtack Jung, Seungho Yoo, Woong Gyu La, Dongkyu Roy Lee, Mungyu Bae, Hwangnam Kim

**Affiliations:** School of Electrical Engineering, Korea University, Seoul 02841, Korea; skylover89@korea.ac.kr (J.J.); pen0423@korea.ac.kr (S.Y.); juhgiyo@korea.ac.kr (W.G.L.); roylee6315@korea.ac.kr (D.R.L.); nardyen@korea.ac.kr (M.B.)

**Keywords:** surveillance, UAV, fleet control, UAV network

## Abstract

Most surveillance systems only contain CCTVs. CCTVs, however, provide only limited maneuverability against dynamic targets and are inefficient for short term surveillance. Such limitations do not raise much concern in some cases, but for the scenario in which traditional surveillance systems do not suffice, adopting a fleet of UAVs can help overcoming the limitations. In this paper, we present a surveillance system implemented with a fleet of unmanned aerial vehicles (UAVs). A surveillance system implemented with a fleet of UAVs is easy to deploy and maintain. A UAV fleet requires little time to deploy and set up, and removing the surveillance is also virtually instant. The system we propose deploys UAVs to the target area for installation and perform surveillance operations. The camera mounted UAVs act as surveillance probes, the server provides overall control of the surveillance system, and the fleet platform provides fleet-wise control of the UAVs. In the proposed system, the UAVs establish a network and enable multi-hop communication, which allows the system to widen its coverage area. The operator of the system can control the fleet of UAVs via the fleet platform and receive surveillance information gathered by the UAVs. The proposed system is described in detail along with the algorithm for effective placement of the UAVs. The prototype of the system is presented, and the experiment carried out shows that the system can successfully perform surveillance over an area set by the system.

## 1. Introduction

Surveillance systems have always been of a great importance. Typical surveillance systems are implemented with multiple closed circuit televisions (CCTV) installed around the area that requires surveillance. While the traditional method provides highly cost effect method regarding the hardware, it has limitations in many ways. First, they are location bound. With a CCTV, a camera is installed to monitor a location, possibly moving a camera direction is provided at most. Second, it requires significant human labor to install and remove the system. To overcome such issues, we propose AVSS, an aerial video surveillance system that uses a fleet of unmanned aerial vehicles (UAVs). AVSS overcomes aforementioned shortcomings as follows. By adopting UAVs as surveillance probes instead of fixed cameras, the probes can move freely in the sky to adjust the captured region. This provides basic adjustments to the change in target areas. Also, with the fleet platform we designed and implemented, a fleet of UAVs can be deployed and retrieved with a click of a button. Therefore, a short term deployment becomes viable, as opposed to traditional systems.

In AVSS, UAVs, specifically multi-rotors, are used as surveillance probes. Single-rotor UAVs can serve the same purpose, but they are not yet widely popular for the danger it causes with their much larger blade wings. Multi-rotors have performance advantages over other types of UAVs, as well as limitations. As a means to replace CCTV, the video captured by the UAV needs to be still. In many cases, the target area requires constant surveillance, and other forms of UAVs cannot provide such still image capturing. Even capturing the same area will require the UAV to constantly tilt/pan the camera, which will require an image processing technique to keep the camera on the same point, but it is not easily doable on a UAV environment. Furthermore, when a target moves in a specific direction, being quickly responsive or following the target with exact speed may not be doable at all with fixed wing UAVs. On the contrary, these are no issues for multi-rotors as long as the object is not faster than its maximum speed. In this aspect, a multi-rotor UAV is a better fit than most of the other options, including but not limited to, fixed wing UAVs, ground vehicle surveillance, and portable towers.

In AVSS, operator can freely set and modify the surveillance area. Operator can setup and modify the surveillance area by simply moving UAVsto an area of interest. Further, scalability can also be supported by utilizing more UAVs. However, there are limitations for UAVs, especially multi-rotors (From this point on, we will refer to multi-rotors as UAVs, non inclusive of other UAV types), to be addressed for practical deployment. First, the flight duration of a UAV is very limited. A commercial UAV can fly at most 30–40 min. It is also to be mentioned that the 30–40 min is the top end of the flight time, and most commercial UAVs can only fly 20–25 min including the ones we use. The time is shortened when the UAVs are equipped with auxiliary devices. Also, UAVs have limited battery life and computing power. The limited computing power makes expensive computing such as high-level image processing implausible. By using a fleet of UAVs as well as light weight algorithms and storage optimization, AVSS has resolved the above challenges.

We consider the following characteristics as the key features of the proposed system, and our focus is on implementing the characteristics into the surveillance system: deployable with minimal effort without consideration of the surveillance area, easy modification of the surveillance area, and rate adaptation to the network status for video streaming. As briefly mentioned, the existing CCTV surveillance system requires much work on installing and maintaining the system. Using a surveillance system with UAVs, the deployment and maintenance process of the system can be simplified. The installation of the proposed system can be carried out simply by sending UAVs to the surveillance area rather than visiting and installing CCTVs at the site. For maintenance, the proposed system can call in the UAVs, perform maintenance procedures, and resend them to the site. The proposed system allows the operator to easily modify the surveillance area by moving the deployed UAVs to the modified surveillance area. Since the UAVs are connected with a wireless network which tends to experience a dynamic network status, the system must have a means to successfully stream video over the network. The proposed system adopted a video stream rate changing scheme that allows the system to stream video with cognition of the network status and anomalous situation.

The key contributions of the paper are as follows:

- We proposed a new surveillance system which consists of multiple UAVs. Contrary to the CCTV surveillance system, the proposed system can be easily deployed and the surveillance area can be modified at anytime. The experiment shows that the system allows the operator to view multiple images captured from UAVs at various locations simultaneously.
- We present a fleet platform that allows the user to control multiple UAVs simultaneously. The fleet platform connects the UAVs through a multi-hop network and allows users to transmit data over the network. The experiment shows that the fleet platform is able to send mission data to the UAVs and deliver surveillance data from the UAV to the user.
- We present a novel algorithm for deploying UAVs to a mission area. When a mission area is given, the algorithm intelligently selects the position of the UAVs that will cover the mission area as effectively as possible.
- We devised an event detection algorithm that helps reducing the amount of required network bandwidth in video streaming. Based on the possible information a stream has, our algorithm controls the rate of the streaming video.

The rest of this article is composed as follows. In Section 2, the background on current systems of UAV fleets and the UAV surveillance are discussed. In Section 3, a step by step guideline to build the fleet is given, and Section 4 explains the placement algorithm in detail. Then the features of our implementation are introduced in Section 5. The implementation details are not about general UAV fleets, but the features explained are essential to the surveillance. Evaluation including simulations and field experiments are provided in Section 6. Finally, we conclude the paper with Section 7.

## 2. Related Work

In this section, the work that has similarity to the UAV surveillance system are introduced. Many researches have been conveyed in a UAV network area, but most researches lack implementations. According to [1], the definition of a flying ad-hoc network (FANET) is a “new form of mobile ad-hoc network (MANET) in which the nodes are UAVs”. FANET is set apart from other networks with characteristics such as: a higher mobility than any traditional networks, the aerial environment, and importance of connectivity [2]. The characteristics of the network adopted in UAV surveillance system are similar to those of FANET definition. Yet, most researches on FANET are theoretical as they are difficult to implement. As there are countless vehicle ad-hoc network papers but only a handful of papers include implementations, the UAV surveillance system is one of the handful of papers with an actual network implementation.

Many of wireless surveillance systems such as Vigil [3] consist of static nodes. Vigil is a real-time distributed wireless surveillance system with edge computing nodes (ECNs). Vigil’s ECNs locally process each camera’s video data and upload only analytic information with significant video frames to the cloud. While Vigil utilizes wireless resources efficiently, its surveillance area is limited because of the static nodes. To overcome this limitation, we deploy UAVs as nodes of the surveillance system, while providing stable network connections with UAVs and users. Li et al. tried to provide consistent video streaming over HTTP [4]. To realize the consistent streaming, authors state that consistent video streaming requires a rate adaption algorithm.

In the work by Choi et al. on CCTV evaluation index [5], the authors devised a method to evaluate the quality of the CCTV image. The index is mainly represented as the ratio between the actual length of an object and its projected length on the image. We have taken out some factors in the work that are not applicable in our work and incorporated camera quality for the index. Nigam et al. conducted a full, comprehensive survey of multiple unmanned vehicle surveillance [6]. This comprehensive survey of the field provides a full background information on video surveillance, and it states the application of ad-hoc network to a persistent surveillance is limited. However, we have built our system on an ad-hoc network while providing persistent network with video streaming service. Our work has network traffic control and video rate adaptation algorithm, which helps the application of otherwise difficult ad-hoc networking. For UAV surveillance, many issues exist: camera angle, UAV mission planning, image stabilization, and more. Semsch et al. proposed control mechanism for UAV-based surveillance in complex urban area [7]. Geng et al. considered a gimbaled camera which has capability of pan and tilt for UAV surveillance and solved the problem of mission planning [8]. Buyukyazi et al. suggested real-time aerial image stabilization system [9]. With the system, the ground station receives data and video stream from the UAV, and conducts the stabilization process. Our work do not include above features, but it can adopt the above and improve overall performance.

## 3. Design of the Proposed System

**Figure 1:**
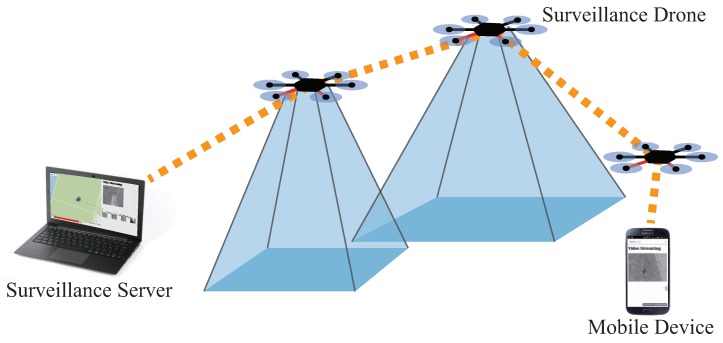
A simple component representation of AVSS.

**Figure 2:**
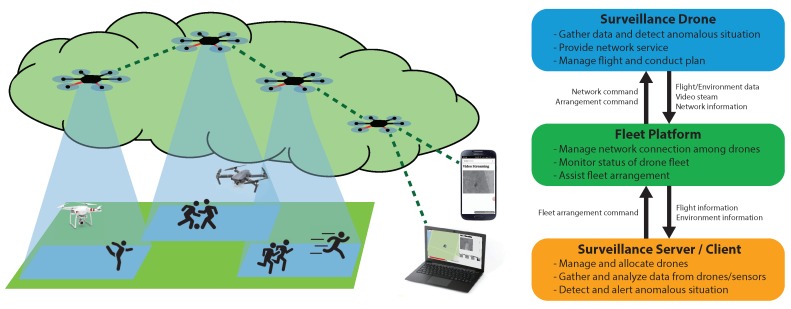
The structure diagram of AVSS.

The purpose of this section is not only to provide a detailed description of the system design, but to give an instruction to build the system from available parts. Here, our guideline to build a fleet starts with the instructions to build a programmable UAV. Three main components of the system are the UAVs, a server, and the fleet platform. The fleet platform is a software we developed to integrate multiple UAVs for an easier management. The concept of the UAVs and the server is both hardware and software, which will be fully covered in this section. In short, the UAVs act as probes that collect information of a designated area of interest, the server gathers, processes, and presents the information from UAVs, and the fleet platform provides a network and management. Overview of the proposed system is shown in Figure 1. As shown in the figure, the server and the UAVs establish a network through which the data is delivered. The UAVs create a multi-hop network, which allows an extended range of command delivery from the server to the UAVs, and vice versa. A usual surveillance scenario of our system is as follows. First, the user inputs mission details, including the mission area, the duration, and the number of UAVs to be deployed, into the surveillance server. After that, the surveillance server processes the mission details into smaller missions for each UAV. The mission for each UAV is passed to the fleet platform to perform soundness check of the mission. The fleet platform delivers the mission to the UAVs through the network of the fleet platform. The UAVs are dispatched afterwards, and they perform the mission provided by the server. During the mission, the data from the UAVs is delivered to the server. Fleet platform resolves problems that may occur during flights, which can be both a mechanical failure or an event worth noting: examples are a battery shortage or an video anomaly. The structure of the system is shown in Figure 2. The figure illustrates the connection between the three components of the system and the modules that consist the components.

### 3.1. Surveillance UAV

Although it is easy to find a manual to build a DIY UAV online [10], a further instruction on building a fleet of programmable UAVs is not easy to find. We provide the manual of building a UAV in this section, as well as the functionalities of it. There can be a variety of methods and designs that can be chosen from, and we tried to choose more major options whenever possible for the easier availability of the related information and technical supports.

Flight controllers such as Pixhawk series or Snapdragon Flight provide offboard control functionality, which means commands from software off the flight controller board can be received and performed. We use offboard functionality because it is not practical to program flight controllers to conduct any complex operations due to its limited capacity. In case of Pixhawk, the offboard messages can be delivered through MAVLink protocol [11]. MAVLink messages can be delivered to the flight controller through a wireless link from the GCS or a wired link from a companion board, which is then also connected to the GCS. A companion board is a microcomputer that is mounted on the UAV, conducting operations that are too expensive for the flight controller to handle. For instance, image processing techniques are too resource demanding for a flight controller. Also, companion boards with autopilot have wired connection to the flight controller, providing robustness against network problems. As it is of high importance that the flight controller performs real time attitude control, it is neither practical nor desirable to implement additional functionality on the flight controller. Therefore, most non-basic operations require a companion board and/or a high throughput wireless link. Even when the fault tolerance is not considered, delays and throughput limitations of wireless link may prevent an agile maneuver of the UAV, and this may cause rejected offboard control in more severe cases because offboard control has a deadline.

For a companion board, most microcomputers that support USB or UART can be used, and we selected Odroid series. For a companion board to communicate with a flight controller, a translating software is required. For MAVLink, MAVROS [12] or MAVProxy [13] can act as the software command delivery system. They are separate processes that help controlling MAVLink-based robots. They are equally viable programs that can convert high-level commands to MAVLink messages. Once these are set up, the UAV with high-computation capability is ready.

In our design, companion board needs to be mounted on a UAV for a few core functionalities: a multi-hop communication, simple image processing, video storage, and video streaming with rate adaptation. The specifics of the fleet will be discussed in next subsection. An aerial network may increase the available bandwidth of the wireless medium by not sharing it with the ground entities, but a UAV needs to be able to communicate with the ground objects, which is carried out with two antennas. Most antennas used in commercial products are omni-directional dipole antennas, which are sufficiently *omni-directional* in a traditional network, but the doughnut shaped radiation becomes *directional* in a three dimensional network as it does not propagate toward the *z* axis of the antenna [14]. In short, two directional antennas are used to avoid the problem: one for vertical communication and another for horizontal communication.

### 3.2. Fleet Platform

Fleet platform provides network and acts as a translator between the server and the acting UAVs. The server can also be called ground control station (GCS), because it is stationed on the ground for the operator. Fleet platform has some portion implemented in the UAV and some in the server, but it is separately described because each of the network and translation functionalities need to be explained together. The general description of the fleet structure is provided in our previous work [15], and the modifications made are as follows.

As shown in Figure 1, fleet platform works as a middleware between the surveillance server, i.e., GCS, and the surveillance UAV. An ad-hoc network supports the fleet platform because multi-hop capability was required for the scalability and wider a mission range. The routing protocol we use is a centralized distance and network status-based GCS-routing, which is elaborated in our previous work [15,16]. Yet, network security is critically impaired with ad-hoc network because most commodity hardware is not equipped with a hardware module for WPA/WPA2 encryption, which drops the throughput down to 10%. Thus, we use WEP security although it is less safe as a trade-off. The discovery of UAVs is carried out using UDP broadcasting. This is an important feature as UAV network can always be attacked and down for an arbitrary period of time, which then requires automatic discovery to repair the network. Traffic flow control is mainly for the multimedia stream as commands and flight information are not heavy enough to create a traffic flow. The server works as a DHCP server and assigns an IP address to each UAV in the fleet.

The coordination and translation of messages are also made in the fleet, so that the drone can operate under the command of the server/operator. First, an operation that has high chance of connection failure is rejected. GCS-routing protocol predicts which topology may be broken; hence the connectivity is thus ensured for deployments. As a technical detail, each UAV has its own coordinates, which is called *local positions*. Server, on the other hand, holds *global positions* as its coordinates, because it needs to manage all UAVs. Therefore, the location commands such as movement or hovering need translation from the global position and local positions. The rate of local position and the attitude information update is 250 Hz, and the global position information is updated 5 times each second. When the connection is broken, the UAV tries to connect back to the server for 10 to 20 s while hovering in the same location, and moves back to base. If it reconnects, it stops going back and waits for the command. When the connection fails, GCS waits for reconnection.

### 3.3. Surveillance Server

Surveillance server directly interacts with the user to set mission and provide necessary information regarding the mission. The user coordinates the mission by setting the surveillance area and the number of UAVs to be deployed. When the user inputs the surveillance area and the number of UAVs to be deployed, the server calculates the individual placement of the UAV using the UAV placement algorithm. Details of the UAV placement algorithm is provided in Section 4.

The server performs supportive but critical functions for the fleet. First, one of the critical limitations of a multi-rotor is the short flight time. This can be overcome by the rotation of the UAVs, which is conducted by the server. When a UAV has a low battery, the server reads the battery status and considers the UAV faulty. All faulty UAVs are removed from acting UAVs, and replaced with another UAV if any other is available. If none is available at the moment, the placement algorithm, which will be discussed shortly, relocates UAVs in the high-priority-first manner. All placement algorithm is run on the GCS, because GCS has much higher computational capability for the lack of weight and size limitations.

## 4. UAV Placement Algorithm

**Figure 3:**
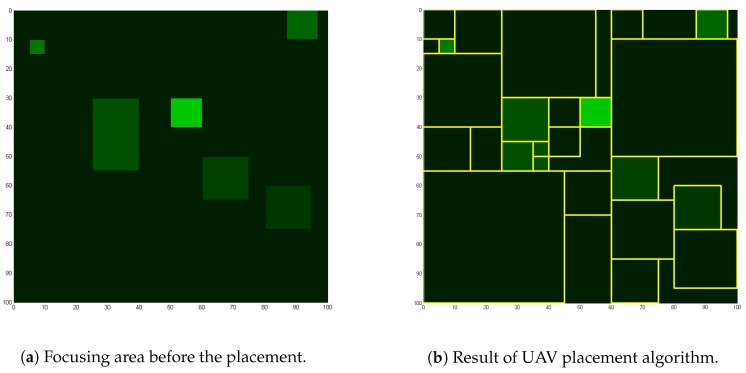
The map of focusing area and UAV placement before and after all placements of 45 UAVs.

In this section, the placement algorithm is explained. The metric to determine what is a good placement and how to find the best placement under such condition is discussed. Figure 3a shows the map of focusing areas before the placements, and Figure 3b is the outcome of placement algorithm which will be explained in this section. Brighter regions mean higher focusing score, and the lines in Figure 3b dividing areas are the boundaries of paced UAVs. The algorithm itself works on the frame level resolution. In other words, this algorithm operates whenever there is a change in the score schematics.

A more important issue is the physical coupling of a target area and the UAV. If an area is completely decoupled with the assigned UAV, the UAV may be commanded to travel a long distance with every frame, which may result in more downtime of surveillance. The design of our algorithm supports a level of physical coupling between a target surveillance area and the UAV assigned to it. Because we use the priority scores of UAVs and the UAV ID, the UAV assigned to a target area will stay assigned to the same target area, even if the target area moves. This ensures the physical coupling of a target area and the UAV unless there is a change in the fleet membership or a change in the order of priority scores. One cycle of our algorithm with 20 UAVs and 15 focusing areas on a laptop, LG 15U780, took an average of 15 ms, which can ensure 66 frames per second, which is fast enough to be considered real time.

### 4.1. Placement Score Metric

The deployment of the cameras plays a critical role in surveillance systems. Because our system is an improvised mobile surveillance infrastructure, an automatic algorithm is much more preferable to manual deployment, which requires human labor. Deploying as many UAVs as possible will always make surveillance more effective, but when the number of UAVs is limited, efficient deployment is crucial to achieve maximum efficacy. Therefore, we have devised a novel algorithm that can automatically deploy UAVs to form a surveillance system based on the information given by the administrator. In this section, the algorithm and the model the algorithm is operating on are explained in steps. The operations, evaluations and analysis of the model will be given in later section. Each region has different degree of importance, and varying importance of region requires different level of attention. We have assumed all regions and camera placements are rectangles that are perfectly aligned with the x,y axes. Camera images are circular, but the edges of a camera capture are distorted so that they are improper for surveillance. We assume the captures are rectangular as rectangles are the end results of most camera captures. In this paper, the region with higher importance is notated as focusing area. The importance of an area is expressed with a score, focusing score. A camera’s quality is expressed as UAV score, which is proportional to the number of pixels in a camera image. The score of a camera placement is expressed as:

(1) $$F\times \left(\frac{D}{h^{2}}+\alpha \right),$$

where *F* is focusing score × area of the intersecting region, *D* is the UAV score, *h* is the altitude of the UAV, and α is the coverage score. The UAV score is inversely proportional to $h^{2}$ because the number of pixels per distance is inversely proportional to the altitude of the UAV, and α is to ensure UAV covers larger region if there is no score difference or it is less significant. Our algorithm places a UAV that maximizes the global sum of the score of each UAV placement. When placing multiple cameras, it is generally better to avoid duplicate placement, but not always. Avoiding redundancies of captures can be useful, but having redundancy in much more important region may be preferable to capturing completely insignificant location. Therefore, Equation (1) is generalized for multiple placements as,

(2) $$F\times \left(\sqrt[{k}]{\sum \bar{D^{k}}}+\alpha \right)$$

for the intersecting camera regions, where $\bar{D}$ is $D/h^{2}$. In the perspective of a new UAV to be placed, the equation acts as an incentive to avoid overlaps, because the score of new UAV means subtraction of the score of previous overlapping UAVs as in,

$$\begin{array}{c} S_{n}=F\times \left[\left(\sqrt[{k}]{\sum_{m=1}^{n}\bar{{D_{x}}^{k}}}+\alpha \right)-\left(\sqrt[{k}]{\sum_{m=1}^{n-1}\bar{{D_{x}}^{k}}}+\alpha \right)\right], \end{array}$$

when k=1, each placement acts as if there is no other UAV placed in the system. In Equations (1) and (2), *F* is the area of coverage with the area score. The coverage of a camera is proportional to the square of the altitude of the UAV, *h*. In other words, *F* is inversely proportional to $\bar{D}$, which makes different sized coverage of the same scored focusing area always has the same incentive. The coverage coefficient alpha acts as the incentive to cover a larger region within the same scored region. In summary, our algorithm deploys UAVs under given focusing scores of each region and global coverage coefficient alone. Because many countries have legal limitations such as no-capture or no-fly zones for UAVs, we devised a method to support such features with our system. In our algorithm, a no-capture or a no-fly zone can also be set by giving a region a large negative focusing score, which will result in UAVs placed around that region. Because it depends on the magnitude of the value and the surrounding focusing score, a substantially large negative focusing score will counter all this.

### 4.2. Placement Search Algorithm

Finding the best placement requires excessively large computation which greatly increases with higher resolution. A placement is composed of three independent variables:x,y coordinates, and the altitude. The *x* and *y* coordinates are the coordinates of the starting point of the rectangle, which is the bottom left corner of the rectangle. Unit width, height, and the score of a rectangle are fixed values per camera, and the height, width, and the score of the placement can be expressed as: unit height × altitude, unit width × altitude, and unit score × altitude${}^{2}$, respectively. It is important to notice that fixed values are for cameras and the values varying on altitude, *h*, are for placements. Each location needs to be evaluated with the sum of scores of all intersecting cameras and focusing areas. In this section, the placement method that has much less computation is explained with a proof. New camera placement requires two step search: the location and the size. The metric, Equation (1), is not dependent on the location of placement as long as the focusing score is constant. Therefore, it is always better to cover a larger area within the same focusing area as we designed. To have a maximized region, *x* and *y* needs to be the bottom left corner of the largest rectangular space within the focusing area. The possible candidates of starting points solely under such assumption is the starting points of all focusing areas, and $\left\{\left(A.x_{e},B.y_{e}\right)|A.x_{e}<B.x_{e}\mathrm{and}A.y_{e}>B.y_{e}\right\}$, where *A* and *B* are focusing areas and $x_{e}$ is the *x* value of the ending point of the rectangle. When a focusing area is within another focusing area, the candidate can also include the starting point of the larger focusing area as the possible candidates. Further, the combination of starting point $x_{s},y_{s}$ also needs to be considered because they may have a larger capture that has maximum score improvement. In this way, the number of possible candidates is $N^{2}+3N-2$, which considers the general case of focusing area deployment where the entire area is $F_{0}$ and others are all within $F_{0}$. The size is dependent on the altitude of the UAV, which also affects the score of the placement. There is no local minima or maxima so that only the size that intersects with other focusing areas or UAV camera needs to be examined. There are three cases of placement: one side increase, both sides increase, and fixed. More specifically, the placement score is expressed as: $S\left(h\right)=F_{1}wlh^{2}\left(\frac{D}{h^{2}}+\alpha \right)$, when both *w* and *l* are increasing with *h*. In this case, $\frac{d}{dh}S\left(h\right)=2F_{1}wlh\alpha$, which is always positive, so we know that there is no local maxima. When *l* is outside the boundary of the $F_{1}$, S(h) is expressed as:

$$\begin{array}{cc} S(h) & =F_{1}^{\prime }wy_{1}h\left(\frac{D}{h^{2}}+\alpha \right)+F_{0}wlh^{2}\left(\frac{D}{h^{2}}+\alpha \right),\\ \frac{d}{dh}S\left(h\right) & =-F_{1}^{\prime }wy_{1}\frac{D}{h^{2}}+F_{1}wy_{1}\alpha +2F_{0}wh\alpha ,\\ \frac{d}{dh}S\left(h\right)h^{2} & =2F_{0}wh^{3}\alpha +F_{1}wy_{1}\alpha -F_{1}^{\prime }wy_{1}D, \end{array}$$

As we know, a cubic function can be expressed as $\left(h-a\right)\left(h-b\right)\left(h-c\right)=h^{3}-\left(a+b+c\right)h^{2}+\left(ab+bc+ac\right)h-abc$. Because we know all coefficients in the above equation are positive, we can observe that a+b+c<0,abc>0, and as *h* is an altitude, h>0, which means there can be only one solution which is a local minima due to always positive $\frac{d^{2}}{dh^{2}}S\left(h\right)$ as long as $F_{1}^{\prime }$ is positive, which is true. Fixed means the derivative is 0, so there is no change in the component. It is to be noted that the iterative deployment algorithm does not guarantee globally optimal placement of UAVs. Globally optimal solution requires much more excessive search, which is impractical. As an attempt to find a better sub-optimal solution, we have tried multiple rounds of iteration over a fixed number of UAVs, but found that simply sorting the UAVs in the order of Dwl makes the algorithm finds the best solution. As an additional merit, sorting the UAVs based on the performance and the focusing areas on the focusing score makes the system always monitor high-priority regions whenever possible. In other words, if there is enough number of UAVs, there is no downtime for any of focusing area. Even when the number of UAVs is insufficient, high priority areas are monitored with no downtime. After a round of placement iteration, each UAV camera is stretched to fit the size of uncovered area if it is mostly desired. Also, if multiple cameras are capturing the same region, the region is divided to each of the camera to maximize the score and effectiveness of surveillance.

## 5. AVSS Features

The native implementation is insufficient as the performances of the commercial products are not powerful enough to support all required functionalities concurrently in real time. Optimizations are made through video compression/selection, network flow control, and other techniques. As video data is the main source of network traffic, we use a compression algorithm to first minimize the required bandwidth, then use weight-based algorithm to allocate the network resource to the most demanding video stream. The compression and selection are carried out based on the event detection algorithm based on an optical flow technique. The use of optical flow may seem as if it is affected by the movement of the UAV. However, as we subtract the average movement vector from the image, the UAV’s movement does not affect the event count.

### 5.1. Event Detection and Video Quality Selection Algorithm

Due to the limited storage of the UAV, storing the video in maximum quality throughout the mission is inefficient. Further, a typical surveillance system, overwrites the oldest data without consideration of its contents when the storage is full. However, most of captured images are unnecessary with no event, so intelligent data storing can greatly improve the storage usage. Our algorithm detects events in the scene and stores the data based on the event metric.

Considering the limited computational power, the event detection algorithm uses optical flow, which is computationally cheap. Algorithm 1 depicts the procedure of the event detection and the video quality selection. F(t) indicates the frame image at *t*, OF(t) indicates the optical flow values of F(t), $\bar{OF(t)}$ indicates mean for optical flow vector from each pixel, δ indicates a threshold value for a pixel to have an event, γ indicates the minimum number of pixels for a frame to be an event, and VQI indicates the visual quality index value for the compression rate of the frame. As a preprocessing step, the algorithm normalizes each vector by subtracting the mean value from all vectors, to compensate the UAV movement because the horizontal movement, tilt, or span is going to cause all pixels to have the same directional vector. Vertical movements are rare unless the UAV is assigned to a new target area with different size. Afterwards, the module checks the optical flow value to see if any event is present. If a predefined number of optical flow values are greater than a set threshold, the module considers the frame to have captured an event. According to the result of the event detection, algorithm calculates the visual quality index of the frame. When consequent frames have no detected events, the video quality index value is slowly decreased. When an event is detected, the video quality index value is increased in a much greater degree to quickly respond to the event.

**Algorithm 1** Event detection and video quality decision.
1:OF(t)← Calculate optical flow between F(t) and F(t−1).2:
$OF\left(t\right)\leftarrow OF\left(t\right)-\bar{OF(t)}$
3:
**for all** (x,y)∈OF(t) **do**
4:    **if** OF(t).x>δ or OF(t).y>δ **then**5:        event_count←event_count+16:    **end if**7:
**end for**
8:**if** event_count>γ **then**9:    Event detected.10:    VQI←VQI+2011:
**else**
12:    Event not detected.13:    VQI←VQI−114:
**end if**
15:
**if** VQI>100 **then**
16:    VQI←10017:
**else if** VQI<10 **then**
18:    VQI←1019:
**end if**



### 5.2. Video Management (UAV)

The video management is essential because of the limited resource: storage, computing power, and the wireless network bandwidth. When there is enough space, there is no downside of storing a video. However, with a limited resource, we remove less important videos when more space is needed. First, 20% of the storage is cleared whenever the storage reaches over 90% of its maximum capacity, from the oldest. If the video’s event score is more than three times higher than the average score of removal candidates, the video is marked as important and untouched. The marked videos are omitted from removal candidates. If it is impossible to remove 20% of the remaining videos when the maximum storage is used, the UAV comes back to base. However, it is extremely unlikely, because average SD cards can store tens of hours of videos.

Because the computing power is also limited, inter-frame encoding schemes are not feasible. Yet, surveillance capability of the UAV requires real-time streaming of the video to users. Therefore, we have employed frame level compression schemes, a web-based implementation with motion JPEG (MJPEG). Each frame is compressed using the event detection scores. As the video capture can be only accessed with one process at a time with MJPEG server, sub-streams of the capture are created. The sub-streams can be sent to the users accessing the stream or saved in the storage. Optical flow-based event detection is implemented here. Each stored video’s duration is 60 s.

### 5.3. Video Rate Adaptation (Fleet Platform)

The users or the administrator should be able to inspect the area even when an abnormal situation is detected by the system. Therefore, the UAVs should have the ability to provide video stream to the users or administrator on request. However, since the bit rate of a video is quite high, we employed a video rate adaptation to fully utilize the network.

The surveillance module reports the video stream information to GCS whenever there is a request of video stream service. Therefore, we can get an entire topology of the networks including the route of video streams in real-time. Based on the information and the positions of UAVs, we can estimate a capacity of each link from the distance [17]. In addition, the video streams are prioritized based on the event score. We consider a set of video requests x=1,…,N and links 1,…,L Let the capacity of link be *i* is $C_{i}$, and a feasible set of rates of request *x* is $v_{x}$. Additionally, the priority is denoted with weights $w_{ij}$. The sum of the weights of all video streams is following:

(3) $$w_{i}=\sum_{j}w_{ij}.$$

Then we divide $v_{x}$ by $w_{x}$ and get $v_{x}^{\prime }$. We adopt a max-min fair (MMF) allocation [18] with weighted value of rate, $v_{x}^{\prime }$. MMF allocation defines that a vector of rates ${v_{x}}^{\prime }$ is max-min fair if no individual rate $v_{x}^{\prime }$ can be increased without decreasing any other rate equal or smaller. We modified progressive filling procedure to find a set of values of $v_{x}$ as follows:

**Step** **1** $v_{x}^{\prime }\leftarrow 0$ for all x∈N,
**Step** **2** Increase all $v_{x}^{\prime }$ at the same speed for all demands, until some link saturated; $c_{l}=\sum_{x}v_{x}^{\prime }$, for all *x* passing by link $L_{l}$,
**Step** **3** Remove saturated links, *l* from **Step 2**, and demands using those links,
**Step** **4** Go back to **Step 2** until no demand left,
**Step** **5** Set the rate $v_{x}$ by multiplying corresponding $w_{x}$ to obtained rate $v_{x}^{\prime }$.

In short, the video rate of each stream has a guaranteed fairness from the centralized control.

### 5.4. UAV Coordination

The coordination of UAVs is managed by the GCS. GCS sends global position request to each UAV consistently. Request-reply method is used instead of simply continuous updates because this method allows both the UAV and the GCS to know when the connection is broken. The main target of management of UAVs is the positions. Using the deployment algorithm discussed earlier, GCS sends each UAV where it needs to be. The identification of the UAVs can be conducted with index and IP. The index of a UAV is much like the index of an element in an array list in Java, changing whenever the membership changes. Index method is useful when the importance job priority is highly significant and the high priority jobs must not be disrupted. The IP of a UAV is unchanged even under network failure. UAV comes back to base when the connection is broken while trying to connect to the GCS. IP-based identification turns the UAV back to the previous mission area.

### 5.5. Data Collection and Presentation

**Figure 4:**
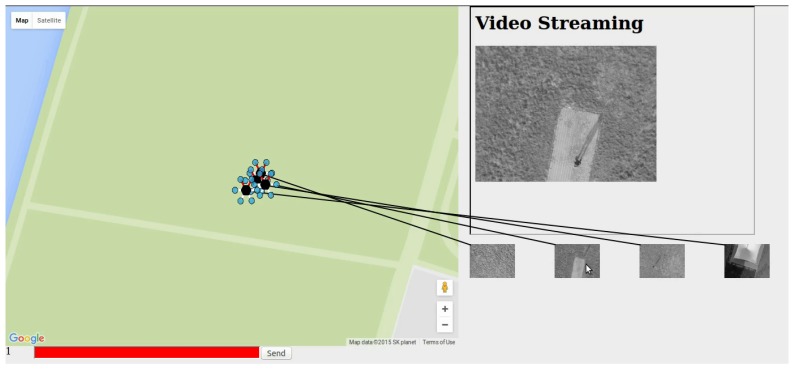
Prototype of Ground Control Station.

**Figure 5:**
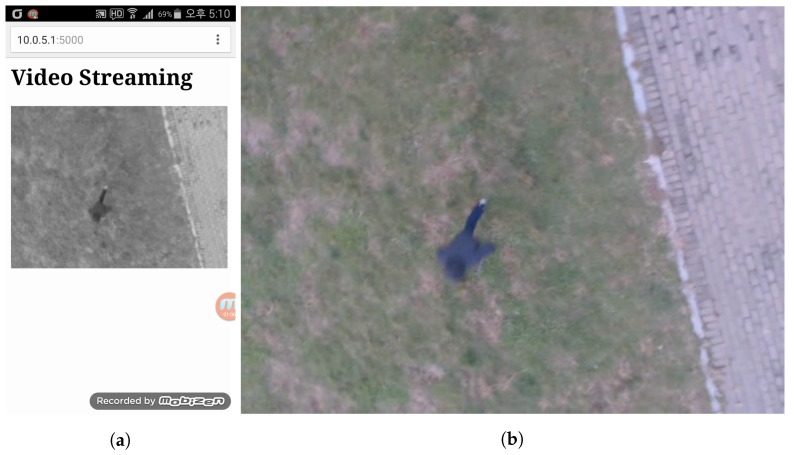
Prototype of the mobile viewer. (**a**) Mobile version of the viewer. (**b**) The video stream coming fome the UAV.

Each UAV has a web server to enable outside access to its information. The web server has a video stream, streamed videos, and an image. The image is the camera’s view which is updated every second. The server collects the still images and shows it to its own web server. The UAVs are only connected to a private network, so GCS downloads the image and shows the image from the local storage from UAVs to allow any user that has access to the GCS can see the pictures. With the web page, the server shows the position of the UAVs and picture taken by the UAVs. Figure 4 shows the prototype of ground control station. In addition, Figure 5a,b shows the mobile view of video server and the streamed video from the surveillance UAV respectively. With Figure 4, we can recognize the location of UAVs, pictures taken by UAVs, and video provided by a selected UAV. The number represented at the left-bottom shows the index of UAV which provides the live video streaming. Also, with the command bar, which represented red text box at the left-bottom, we could send movement command to UAV.

## 6. Evaluation

The simulations and empirical experiments are given in this section. We have conducted a series of simulations on the placement algorithm. The empirical results from the experiments we carried out are also given in this section.

### 6.1. Simulations

In this section, the placement algorithm we devised is evaluated in various ways. First, the global and local score change along with increasing number of UAVs is provided. The time consumed to obtain each placement is also discussed, and the effect of different α value is shown.

#### 6.1.1. Number of UAVs

**Figure 6:**
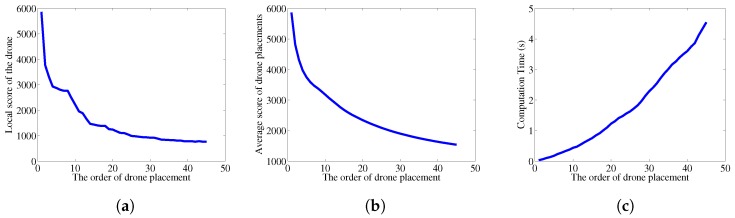
The metric values with respect to the number of UAVs. (**a**) the local score. (**b**) the average score. (**c**) the computation time.

Figure 6 a,b are the presentation of placement scores. Figure 6a shows the global score increments of each placement, thus local score. In all graphs, *x* axis is the number of UAVs that have been placed, and *y* axis is the score. Focusing area scenario is shown in Figure 3b, which has 7 focusing areas. It can be observed that the local score drops significantly at 8th UAV, which shows that the efficiency of placement more than the number of focus area is considerably less efficient. Our algorithm does not include any external measures to limit the number of UAVs even when more is available, but other metrics such as the energy or the noise can be factored in to find the optimal number of UAVs to be placed. Considering only surveillance quality, more UAVs are always better.

#### 6.1.2. Time Constraint

Figure 6c shows the time consumed to find each step of placement. Because each score calculation has to consider the UAVs that has been deployed so far, the time consumed to find a placement location and altitude linearly increases. Thus, the time consumed to deploy *n* UAVs has time constraint of $n^{2}$. However, the case of deploying 45 UAVs is extremely exaggerated, especially when the purpose of the algorithm is to find the sub-optimal solution for a small number of UAV deployment. Even under such extreme condition, time to place a UAV takes less than 5 s.

#### 6.1.3. α Value

**Figure 7:**
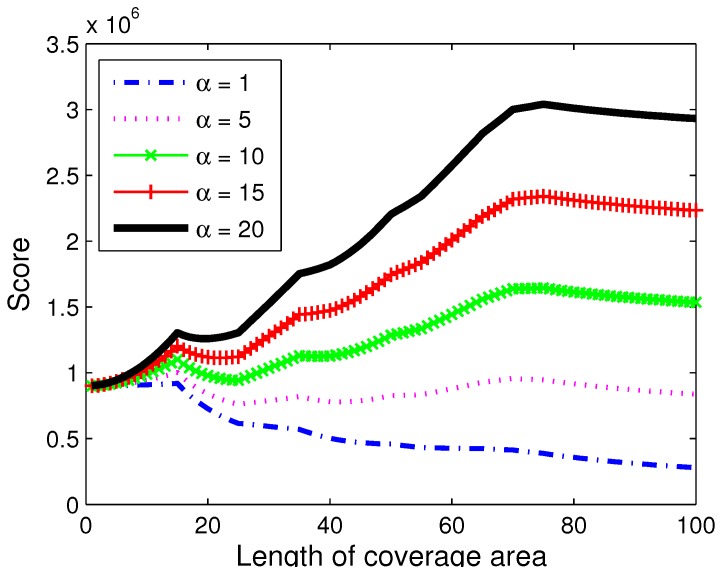
Score with varying α value.

It is introduced in the previous section that the α value of our model is designed to give incentive to larger coverage over smaller coverage. Figure 7 shows the placement score depending on the α value. *x* axis is the width of the placement, and *y* axis is the placement score. A square shaped placement is used. As can be seen, the effect of placement width is almost insignificant when alpha is small. However, larger α value increases the overall placement value as the width grows. It can be observed that lower α value makes smaller placement focused on the focusing area more likely to be selected, while larger α value makes the algorithm prefer larger scale capture.

### 6.2. Experiments

In this section, we discuss the experiment of our proposed surveillance system. To verify the functionality of the system, we plan and conduct experimental scenario. In the scenario, surveillance server plans the mission with given focus areas. After the mission is prepared and uploaded to the UAVs, the UAVs take off from the base camp, move to the points where surveillance server planned. After UAVs reached the points, then the UAVs maintain the position, collect information, and send the information to GCS. The GCS collects information from UAVs, which conduct missions, and visualize the collected information to provide information to the user.

**Figure 8:**
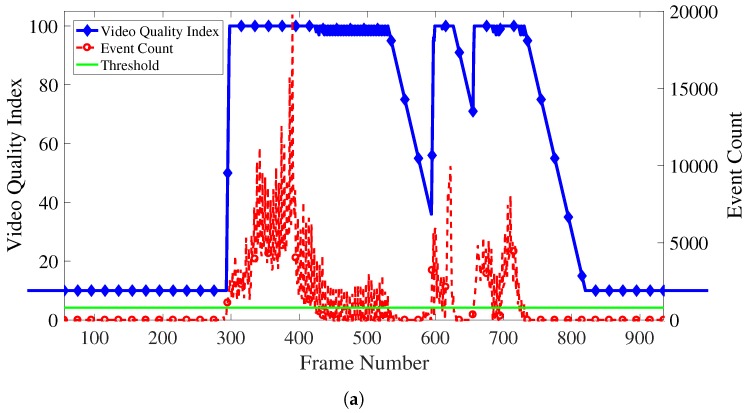

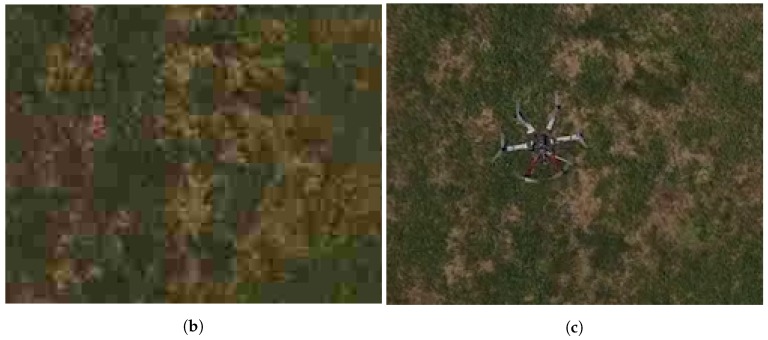
Varying bit rate of video streaming: (**a**) Varying VQI based on the anomalous degree of the video frame; (**b**) Frame with low anomalous degree in a low quality video (frame 107); (**c**) Frame with high anomalous degree in a high quality video (frame 609).

We conduct an experiment with 4 UAVs, surveillance at about 15 m and 25 m above the ground level, continuously collects visual information from the point. Throughout the experiment, UAVs periodically send pictures to GCS and the GCS shows the received pictures to the user through the web page. In addition, the user who monitors the surveillance area, views the information provided by the server, and selects the suspicious area. During the surveillance, subjects appeared in the target area. We can see from Figure 8a that the movement was quickly reflected within 2 frames.

**Figure 9:**
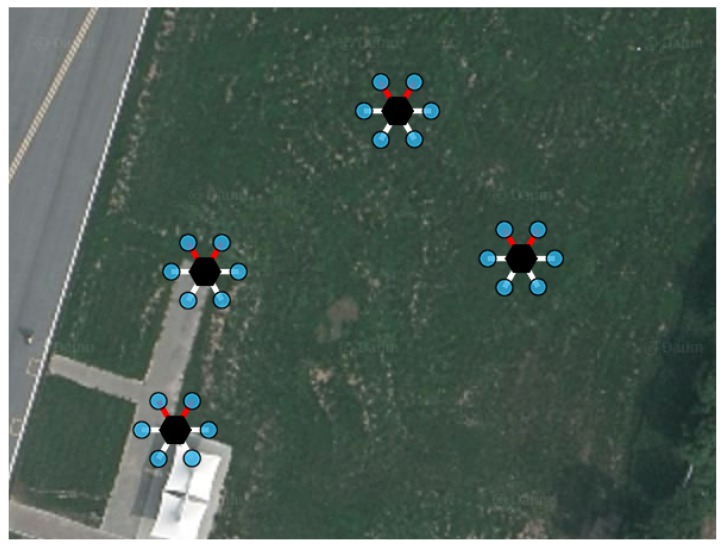
Satellite map of experimental area with position of UAVs.

The satellite map of experiment area and the position of UAVs are in Figure 9. The summary video for an entire scenario of the experiment can be found on Youtube [19].

### 6.3. Variable Bit Rate Video Streaming

Functionality of the variable bit rate video streaming was tested by performing video streaming using the proposed system. The streamed video first starts with no object in the scene, and latter in the video couple of people walk and run across the video. Figure 8a depicts the resulting video quality index values, which are calculated based upon the event detection of the video. As the figure shows, the beginning part of the video starts in low video quality index since no objects is present and therefore, no event is detected. When the event count is above the threshold, the video quality increases accordingly, and it drops slowly when there is no event detected for a while. Afterwards when people start to walk across the video, an event is detected by the system, and the video quality index increases. Figure 8b,c are captured images of the streamed videos. Figure 8b is an image from the beginning part of the video where video quality index is low. Therefore, the quality of the video is poor and the image is visibly pixelated. Contrarily, Figure 8c is an image from the part where people are walking and running in the video. The system recognized the event and, therefore, the video quality index is increased. The resulting video image shows that the quality of the video is much great and the block boundaries are hard to identify.

## 7. Conclusions

We designed and implemented a surveillance system, which based on the networked UAVs. We have integrated hardware, adopted existing algorithms, and devised new algorithms for the allocation of UAVs. We defined the problems and solved them with our design, where three main modules work collectively: the surveillance UAV, fleet platform, and the surveillance server. The UAV-based surveillance system is successfully constructed with our design, and the verification and evaluation are presented. With the surveillance system, UAVs can surveillance together at the surveillance area and have more capability than single UAV. The system does not have only a limited used for simple surveillance, but can also be utilized in the future for surveillance assistance at disaster areas. Also, as a future work, we will be working on algorithms and system designs to inclusively cooperate with other types of UAVs as well as fixed location cameras.
